# Short-Term Intra-Subject Variation in Exhaled Volatile Organic Compounds (VOCs) in COPD Patients and Healthy Controls and Its Effect on Disease Classification

**DOI:** 10.3390/metabo4020300

**Published:** 2014-05-09

**Authors:** Christopher Phillips, Neil Mac Parthaláin, Yasir Syed, Davide Deganello, Timothy Claypole, Keir Lewis

**Affiliations:** 1 Welsh Centre for Printing and Coating, and Centre for Nano Health, College of Engineering, Swansea University, Singleton Park, Swansea SA2 8PP, UK; E-Mails: d.deganello@swansea.ac.uk (D.D.); t.c.claypole@swansea.ac.uk (T.C.); 2 Department of Computer Science, Institute of Maths, Physics and Computer Science, Aberystwyth University, Aberystwyth SY23 3DB, UK; E-Mail: ncm@aber.ac.uk; 3 Institute of Life Science, College of Medicine, Swansea University, Singleton Park, Swansea SA2 8PP, UK; E-Mail: k.e.lewis@swansea.ac.uk; 4 Respiratory Unit, Prince Philip Hospital, Hywel Dda University Health Board, Llanelli SA14 8QF, UK

**Keywords:** COPD, Exhaled volatile organic compounds (VOC), variation, machine learning

## Abstract

Exhaled volatile organic compounds (VOCs) are of interest for their potential to diagnose disease non-invasively. However, most breath VOC studies have analyzed single breath samples from an individual and assumed them to be wholly consistent representative of the person. This provided the motivation for an investigation of the variability of breath profiles when three breath samples are taken over a short time period (two minute intervals between samples) for 118 stable patients with Chronic Obstructive Pulmonary Disease (COPD) and 63 healthy controls and analyzed by gas chromatography and mass spectroscopy (GC/MS). The extent of the variation in VOC levels differed between COPD and healthy subjects and the patterns of variation differed for isoprene *versus* the bulk of other VOCs. In addition, machine learning approaches were applied to the breath data to establish whether these samples differed in their ability to discriminate COPD from healthy states and whether aggregation of multiple samples, into single data sets, could offer improved discrimination. The three breath samples gave similar classification accuracy to one another when evaluated separately (66.5% to 68.3% subjects classified correctly depending on the breath repetition used). Combining multiple breath samples into single data sets gave better discrimination (73.4% subjects classified correctly). Although accuracy is not sufficient for COPD diagnosis in a clinical setting, enhanced sampling and analysis may improve accuracy further. Variability in samples, and short-term effects of practice or exertion, need to be considered in any breath testing program to improve reliability and optimize discrimination.

## 1. Introduction

Diagnosis of lung disease via breath chemical analysis has attracted increasing interest for a range of pulmonary conditions such as lung cancer [1,2,3,4], cystic fibrosis [5], tuberculosis [6], asthma [7] and, the subject of this study, Chronic Obstructive Pulmonary Disease (COPD) [8,9]. However, there is a lack of understanding about the variability in expression of breath VOCs (volatile organic compounds) within each individual and whether this influences the ability to discriminate healthy and diseased states. Therefore, the aim of this study was to examine intra-subject variability of breath profiles when multiple samples are taken for COPD patients and healthy controls over a short time period. Firstly, to see if there is any difference in patterns of variation between healthy and diseased states and secondly, to evaluate the ability of the data from these different breath sample repetitions, both individually and in aggregated form, to distinguish COPD from healthy controls using machine learning techniques.

Other studies, which examined VOCs in the context of various lung diseases, have tended to concentrate on single breath samples (although these may be gathered from multiple sequential exhalations). However, they do not consider short term variability, which may affect breath composition for single exhalations and the changing composition of breath during repeat exhalation. The limited data on intra-individual variability in VOCs in breath has predominantly focused on healthy subjects (reported in discussion) and has not been sufficiently considered in the context of disease state classification. The ways in which the body creates, metabolizes and excretes VOCs has implications for measuring not only healthy physiology but also for interpreting findings in any disease state to be monitored via breath sampling. There are a number of factors to consider:

(1) The relationship between blood and breath VOC
   Accurate sampling is often based on the assumption of partial pressures of VOCs in the alveolar regions in the lung being in equilibrium with the bloodstream. However, the presence of lung disease itself will affect the delivery of breath to the sampler. Chronic obstructive and interstitiallung disease impairs pulmonary gas exchange leading to wasted ventilation (alveolar dead space) and wasted perfusion (venous admixture) and hence the composition of chemicals differs in the sample. Any co-morbidities affecting cardiovascular, hepatic or renal systems will alter how the body generates, metabolizes and excretes chemicals in the body that contribute to breath VOCs. Moreover, changes in even healthy metabolism can occur over the short term [10,11,12].
(2) Ability to deliver a breath sample
   The presence of lung disease will itself affect the ability of the subject to deliver breath to the sampler in a reliable and controlled fashion; particularly from the alveolar part of the lung. Another potential source of variation is the subjective element of the sampling, with a dependence on the effort and individual makes to exhale into the sampler [13].
(3) Response to exertion
   VOC expression varies in response to any exertion (e.g., compensatory increases in heart rate or ventilation: perfusion matching) [14]. This is likely to be different for healthy and diseased states. Subtle changes in lung function and breathing patterns prior to testing may affect VOC expression, whether these are endogenous or exogenous in origin [15]. The act of giving a breath sample may itself cause changes in subsequent samples.

For any diagnostic test to be clinically useful, it should ideally be non-invasive (acceptable to the patient), repeatable and accurate *i.e.*, representative of the individual/disease tested and should not vary appreciably over time, unless disease state changes. If not consistent, the pattern of variation should at least be predictable. A range of breath sampling techniques are available which will be affected by the aforementioned factors in different ways. However, this study aims to highlight effects which are of interest regardless of the underlying sampling methodology. This study therefore investigates the variability of breath profiles when three breath samples were taken over a short time period for 118 stable COPD patients and 63 healthy controls. The focus is on isoprene, a ubiquitous VOC in breath, which behaves differently to most others, and total VOC levels (minus isoprene) to provide an overall view of breath VOC. A range of machine learning approaches were applied to the breath data to establish whether the samples (breath 1, 2 or 3) differed in their ability to discriminate COPD *versus* healthy states and whether aggregation of multiple samples, into single data sets could offer improved discrimination. 

## 2. Experimental Section

### 2.1. Subjects

This is part of a study approved by a local research and ethics committee and registered with International Clinical Trials Network (ISRCTN 82911859). The cohort comprised of 118 patients with COPD and 63 healthy controls (Table 1). Full information on the groups and their recruitment has been published previously and in more detail [16] together with a description of COPD severity.

**Table 1:** Chronic Obstructive Pulmonary Disease (COPD) and control groups in the study.

Variable (Mean ± SD)		COPD (*n* = 118)	Controls (*n* = 63)
Age (years)		67.0 ± 8.4	67.4 ± 9.7
Male		61%	47%
Smoking Status	-never	0	39
	-ex	78	18
	-current	40	6
Body Mass Index (kg/m^2^)		25.6 ± 4.5	27.0 ± 4.4
Predicted % FEV_1_		49.6 ± 18	98 ± 16
Oxygen saturation %		95.0 ± 2.4	95.8 ± 2.3

Smoking status has previously been demonstrated to act as a confounding factor in COPD diagnosis; with a balanced smoking status in COPD and healthy groups leading to significantly improved classification of healthy subjects [16]. This was achieved by eliminating current smokers from the analysis and treating all samples as separate objects (although data from the same subject did not appear simultaneously in the training and testing phases). Machine learning techniques similar to those used in this study (further details in Section 2.5) were employed. In this article, this is not explored as the objective is to evaluate the comparative classification accuracy when using sequential and combined breath data rather than optimizing discrimination. There is also a reduction in the amount of data to learn from when subjects are removed, as well as an increased class imbalance which is exacerbated when splitting the data into sub sections.

### 2.2. Breath Sampling Procedure

The same clinical room (with closed door and no air conditioning) within the hospital was used for sample collection over multiple days. Patients with COPD were identified from hospital registers. All subjects were instructed to fast for 4 h and omit their inhalers that morning (6–8 h before testing). They then performed spirometry (Vitalograph Alpha^®^, Buckinghamshire, UK) in the sitting position to obtain Forced Expiratory Volume in one second and Forced Vital Capacity to confirm obstructive airways disease and the clinical diagnosis of COPD (using guidelines from Global Initiative for Chronic Obstructive Lung Disease—GOLD [17]), or normal lung function. They were then rested for 20 min within the test room prior to breath collection. By remaining in a single room for this time the subject will inhale the surrounding environmental air to allow better equilibrium between alveolar and room air; although there is no clear indication from the literature as to how long a subject should remain inside a room. The rest time also allows the subject to recover from any exertion, due to spirometry and walking to the room, and to return to their resting heart and respiratory rate whilst also maintaining a simple and time efficient methodology.

Each participant provided three breath samples in succession with a two minute time interval between each breath. While a longer interval between breaths might allow equilibrium to be re-established for each test, this time frame was selected for evaluation of short-term exertion effects and relevance for multiple exhalation techniques such as inflation of a Tedlar bag. This time period also allows a short rest period for COPD patients, who may feel breathless and was a convenient time period for the subject.

Breath samples were collected using a commercially available sampler (Bio-VOC^®^, Markes International Limited, Llantrisant, UK). Participants took a deep breath and then slowly exhaled as fully as possible through the sampler to their residual volume. Exhalation rate was not measured but was limited by the resistance offered by the sampler. The sampler consists of a PTFE syringe which holds 129 milliliters of air. The sampler has a 3 mm diameter orifice, at the opposite end to where the breath is introduced, to allow flow through with the aim that the initial dead-space air is displaced progressively by air from lower in the lungs, and eventually the alveolar region, as exhalation proceeds (although there is no data on the breath distribution for these samplers). After complete exhalation, a hand-operated plunger is used to slowly force the breath through the orifice into a preconditioned sorbent tube in which VOCs are trapped (Carbograph 1TD and Carbopack X). The same operator was used to sample all subjects. Several BioVOC samplers were used during sample collection and were flushed with ambient air before and after each sample was taken. The BioVOC sampler was selected for its ease of use both for the operator and the subject. However, a disadvantage of this apparatus is that the exhalation into the sampler is not controlled. VOCs were extracted, separated and analyzed using thermal desorption (TD) and gas chromatography-mass spectrometry (GC-MS). The TD system was an Ultra unit (Markes International, Llantrisant, UK) while the GC was a 6890N (Agilent Technologies) and the MS a 5973 network mass selective detector (Agilent Technologies). Methodology has been described in full previously [16]. All three repetitions from a given individual were processed in the same TD run in batches of 7 to 10 subjects *i.e.*, 21 to 30 tubes. Automated peak detection and baseline correction was used to calculate peak area (area under the curve) for each compound. VOCs were identified using an automated library search function in the Chemstation GC/MS software (Agilent Technologies) coupled with the NIST 98 mass spectral library (The National Institute of Standards and Technology).

### 2.3. Data Normalization

Over the course of the sampling there were drifts in TD cold trap and mass spectrometer sensitivity. Therefore prior to analysis, this had to be compensated for so that overall trends across and between groups could be readily compared and this drift did not influence machine learning outcomes. The normalization methods used for this have been reported previously [16]. A benefit of employing such normalization is that it removes the need for placing internal standards into the samples. Two separate normalization methods were required for the reasons outlined below.

In order to illustrate relative magnitudes of compounds between COPD and healthy states, VOC levels were divided by the average area under the curve for the ubiquitous VOC benzaldehyde in the entire batch of measurements from which it originated (20 or so samples rather than individual samples). This effectively acts as a sensitivity adjustment for the TD-GCMS system over the duration of the sampling and is useful for comparing overall trends across and between groups. Benzaldehyde did vary from sample-to-sample but averages for both COPD and control groups within a given batch were similar across the series of collections as demonstrated previously [16]. Benzaldehyde has also been reported in other studies as being ubiquitous in breath [18,19] with data suggesting an environmental source due to its presence at higher levels than in breath [19,20] which was also observed in this data [16]. Furthermore, Filipiak *et al*. [20] reported identical mean levels of benzaldehyde in a group of non and ex-smokers when compared with a group of current smokers. Individual intra-subject variation did not require any normalization as drifts in sensitivity were not expected during the same TD-GC-MS run.

For machine learning analyses, which were employed for the comparison of the ability of the different breath repetitions to discriminate COPD from healthy status, the total magnitude of VOC levels were used to normalize each sample (*i.e.*, the sum total magnitude of all VOCs in a particular sample). This method is self-contained and not reliant on other samples. Furthermore, data utilizing this approach has been shown to generally perform better in automatic data analysis techniques than benzaldehyde normalized data [16]. Total VOC was calculated for a retention time window that excluded noisy spectra at low retention time and column bleed at high retention time, and with known system peaks omitted [16]. A similar approach has also been used by Van Berkel [7,21] to compensate both for mixing of alveolar and upper airway breath in the sample and drifts in sensitivity of the test equipment. This method is not appropriate for the comparison of differences in magnitudes of VOCs in COPD and control subjects as it effectively reduces the levels of individual VOCs in the COPD group which tends to have substantially higher levels of VOC.

### 2.4. Analysis of Intra Subject Variability in VOC Levels

Levels of isoprene and total VOC (excluding isoprene) were selected as parameters to monitor the variation in breath VOC in the short term. Isoprene was selected as a stand-alone VOC as it was found in all samples and has been shown in literature to vary according to heart-rate and ventilation, in a manner contrary to most other VOCs (for reasons detailed later in the article). Total VOC minus isoprene was used as an indicator of the quantity of the volatile material emitted by the subject; details on its calculation are described in the previous section. Normalization with respect to batch levels of benzaldehyde was used to calculate mean VOC levels in breaths 1, 2 and 3 across all subjects in COPD and control groups.

To further demonstrate the pattern of variation over the three breaths, a range of further VOCs were considered and the areas for the second and third repetitions were divided by the first for each individual subject. Median and geometric means of these ratios were then calculated to demonstrate overall trends of variation in breath VOC. In addition to isoprene and TVOC-isoprene, values were calculated for benzene, toluene, benzaldehyde, hexanal and nonadecane. These are examples of ubiquitous VOCs, which have been previously identified as useful features in machine learning, and should therefore influence the ability to classify diseased and healthy states should they vary [16]. Intra-subject coefficient of variation was also calculated over breaths 1, 2 and 3 (standard deviation divided by mean and expressed as a percentage).

### 2.5. Classification into COPD and Control Groups Using Machine Learning Methods

For the experimental evaluation step, three different types of analysis were performed on the data:

(1) The individual breath samples (1, 2 and 3) were analyzed in isolation for their ability to discriminate COPD from healthy status. That is, all breath 1 samples from COPD and controls were combined into a single dataset prior to analysis. The same procedure was implemented for breath 2 and for breath 3.
(2) Combinations of subsets of data were formed (breath samples 1 and 2, 1 and 3, and 2 and 3) were combined into three different datasets respectively. This means that only the data objects (samples) for the respective breaths were included, with each dataset containing 364 (182 + 182) data objects. Note that data objects relating to the same subject were not allowed to appear simultaneously during the training and testing phases of the cross-validation.
(3) Breaths for each subject were summed to form single data sets for the subject and analyzed for their ability to discriminate (Summing all breaths was found, in preliminary studies, to be the most robust method for aggregating data, with consistency across a large range of classifiers. Other methods trialed included mean, maxima and minima, and ordered weighted aggregation (OWA) [22], which are shown in the supplementary data file. Note that no subjective or *a-priori* domain information was used in any of the aggregation processes).

Classifier learning was carried out based on the full set of VOCs using the Weka [23] data-mining suite. An additional preprocessing step was integrated into the cross-validation phase of Weka in order to exclude data objects (where necessary) that were related to the same subject from simultaneously appearing in the training and testing phases. Three classifier types were used; namely J48, a version of Quinlan’s ID3 decision-tree algorithm [24], JRIP, a rule-based “ripper” classifier which generates rules and uses these to classify “new” objects [25] and PART a rule-based classifier [26]. These have been shown to be effective and robust with this data as well as being resilient to noise in the data [16]. Preliminary data using different classifiers is given in a supplementary data file.

For the training, testing and validation phases, 10 different data randomizations of stratified 10-fold cross-validation (10FCV) for the generation of results. This was performed ten times to give a total of 100 runs in order to give more stable and hence realistic results when compared with a single 10 FCV run. The performance of the classifier learners was characterized in terms of overall classification accuracy of disease state, as well as sensitivity (the proportion of correct predictions for COPD subjects) and specificity (the proportion of correct predictions for control subjects). The area under the Receiver Operating Characteristic (ROC) curve (AUC) was also calculated. Many of the classifier learners offer similar performance, but in some cases also appear to offer an improvement in performance. In order to investigate whether such results are statistically significant, and not merely due to chance, a paired t-test with a significance value of 0.05 was also carried out.

## 3. Results and Discussion

### 3.1. Variation between Breaths 1, 2 and 3

**Figure 1:**
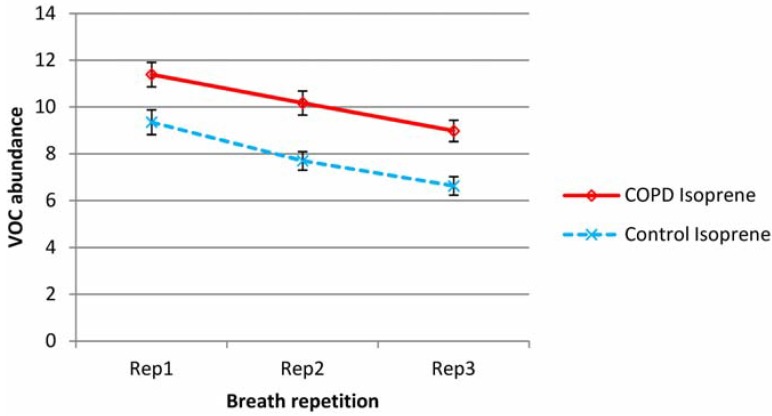
Variation in mean isoprene levels in repeat breath tests for both COPD and control groups (abundance normalized according to batch benzaldehyde). Error bars show standard errors.

**Figure 2:**
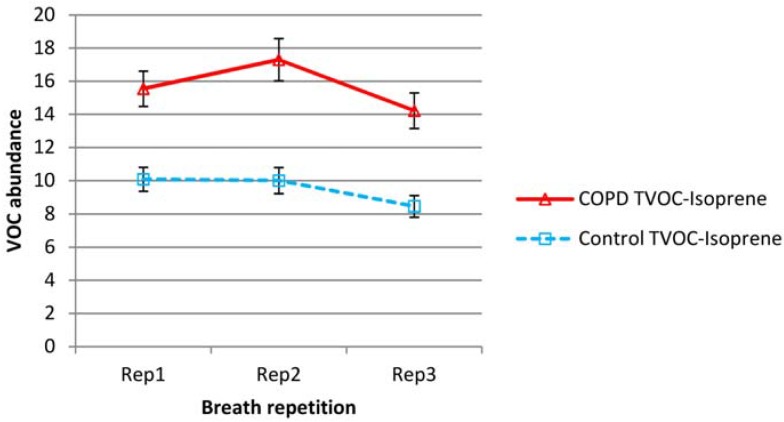
Variation in mean total volatile organic compound (TVOC) minus isoprene levels in repeat breath tests for both COPD and control groups (abundance normalized according to batch benzaldehyde). Error bars show standard errors.

Mean isoprene and total VOC minus isoprene levels over all subjects are shown in Figure 1 and Figure 2 respectively for both COPD and control groups (normalized according to batch levels of benzaldehyde—see experimental section). To remove the effect of outliers (in the graphed data), which had a particularly significant effect on the variability of the TVOC data, from subject to subject, the three lowest and three highest values were removed from all sets of data.

Median values and geometric means of the ratios of breaths 2 and 3 to breath 1, over all subjects in COPD and healthy groups for isoprene, total VOC minus isoprene, benzene, toluene, benzaldehyde, hexanal and nonadecane are shown in Table 2. Mean intra-subject coefficients of variation for the three breaths are also shown. No normalization or omission of outliers is employed for the data in Table 2.

**Table 2:** VOC levels in second and third breath tests compared with first in both COPD and control groups—median, geometric means and coefficient of variation across each group are shown.

	VOC	COPD		Control	
		2/1	3/1	2/1	3/1
Median	Isoprene	0.930	0.750	0.755	0.678
	Total-isoprene	1.143	0.936	1.196	0.705
	Benzene	1.071	1.061	0.982	1.002
	Toluene	1.067	1.027	0.979	0.992
	Benzaldehyde	1.151	1.036	1.036	0.978
	Hexanal	0.973	0.843	0.967	0.860
	Nonadecane	0.995	0.980	1.013	0.989
Geometric mean	Isoprene	0.901	0.752	0.781	0.641
	Total-isoprene	1.093	0.933	1.043	0.836
	Benzene	1.124	1.058	0.979	1.006
	Toluene	1.080	1.023	0.961	0.978
	Benzaldehyde	1.153	0.983	0.950	0.969
	Hexanal	1.036	0.929	0.962	0.827
	Nonadecane	1.002	0.975	0.956	0.897
CV%	Isoprene	23		26	
	Total-isoprene	40		40	
	Benzene	21		23	
	Toluene	18		18	
	Benzaldehyde	26		24	
	Hexanal	21		21	
	Nonadecane	28		27	

Isoprene was a substantial element of breath VOC and was present in higher levels in the breath of COPD patients than controls. In both COPD and control groups, exhaled isoprene levels fell steadily upon repeat testing. Proportionally, this drop was higher in healthy subjects. Total VOC minus isoprene, was also higher in the COPD group but showed a different pattern; for the COPD group there was increased abundance in the second sample and then a reduction on the third sample to levels below that of the first breath. For the control group, there was little difference between breath 1 and 2, but there was a decline in breath 3.

Outliers had little effect on the mean but mainly affected variability. For isoprene, the average intra-subject coefficients of variation (over breaths 1, 2 and 3) were 23% and 26% for COPD and control groups respectively. For Total VOC minus isoprene, the average coefficients of variation were higher at 40% for both groups. Individual VOCs showed no evidence of the substantial reductions seen in isoprene and were more in line with the TVOC-isoprene pattern. Benzene, toluene, benzaldehyde and hexanal levels appeared to rise in breath 2, then fall slightly for COPD patients. Hexanal and nonadecane fell across sequential breaths for controls but benzene, toluene and benzaldehyde did not show any significant change.

### 3.2. Classification of Subjects into COPD and Healthy States Using Machine Learning Methods

**Figure 3:**
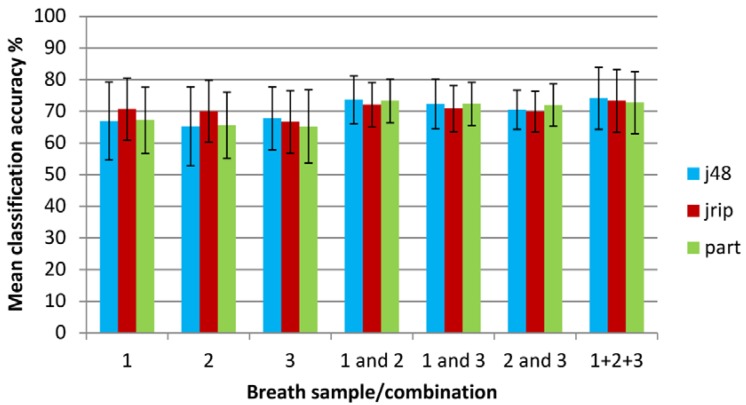
Classification accuracy when using machine learning techniques on individual and combined breath samples (error bars show standard deviations).

Classification accuracies for the different breath repetitions and combined breath samples are shown on an overall basis and individually for COPD and control groups in Table 3. Area under the ROC curve is also shown. The values quoted are the mean accuracies from all runs of 10 × 10 FCV (100 runs) with standard deviations in parentheses. Overall classification accuracies are also compared graphically in Figure 3. The data includes the individual breath samples (1, 2 and 3) analyzed in isolation, combinations of subsets of data (breath samples 1 and 2, 1 and 3, and 2 and 3) combined into three different datasets respectively with each dataset containing 364 (182 + 182) data objects and breaths for each subject summed (1 + 2 + 3) to form single data sets for each subject.

**Table 3:** Classification accuracy when using machine learning techniques on individual and combined breath samples.

Classifier	1 only	2 only	3 only	1 and 2			1 and 3		2 and 3	1 + 2 + 3	
*Overall classification accuracy % (standard deviation)*											
J48	66.91(12.30)	65.28(12.45)	67.75(9.98)	73.66(7.52)			72.30(7.88)		70.46(6.17)	74.13(9.84)	
JRIP	70.69(9.85)	69.99(9.76)	66.64(9.88)	72.11(6.99)			70.85(7.29)		69.93(6.44)	73.28(9.93)	
PART	67.18(10.47)	65.58(10.49)	65.24(11.55)	73.28(6.90)			72.38(6.78)		72.01(6.66)	72.74(9.80)	
Mean	68.26	66.95	66.54	73.02			71.84		70.80	73.38	
*Area under the ROC curve*											
J48	0.67(0.14)	0.65(0.14)	0.66(0.13)	0.72(0.10)			0.72(0.10)		0.69(0.09)	0.65(0.15)	
JRIP	0.66(0.12)	0.65(0.11)	0.63(0.11)	0.69(0.09)			0.68(0.09)		0.65(0.08)	0.70(0.12)	
PART	0.67(0.14)	0.61(0.12)	0.63(0.14)	0.73(0.09)			0.70(0.10)		0.71(0.08)	0.65(0.16)	
Mean	0.67	0.64	0.64	0.71			0.70		0.68	0.67	
*Proportion of correct predictions for COPD subjects (standard deviation)*											
J48	0.71(0.15)	0.69(0.16)	0.75(0.14)		0.80(0.08)	0.79(0.09)		0.78(0.08)		0.69(0.19)	
JRIP	0.81(0.12)	0.81(0.12)	0.76(0.16)		0.81(0.09)	0.79(0.09)		0.81(0.10)		0.75(0.15)	
PART	0.73(0.14)	0.73(0.14)	0.73(0.14)		0.79(0.08)	0.80(0.09)		0.80(0.09)		0.66(0.19)	
Mean	0.75	0.74	0.75		0.80	0.79		0.80		0.70	
*Proportion of correct predictions for Control subjects (standard deviation)*											
J48	0.58(0.22)	0.58(0.21)	0.54(0.20)		0.62(0.15)	0.59(0.15)		0.55(0.12)		0.59(0.22)	
JRIP	0.51(0.22)	0.48(0.19)	0.50(0.25)		0.56(0.19)	0.55(0.17)		0.50(0.18)		0.64(0.21)	
PART	0.57(0.19)	0.52(0.21)	0.51(0.21)		0.63(0.15)	0.58(0.14)		0.57(0.14)		0.62(0.23)	
Mean	0.55	0.52	0.52		0.60	0.57		0.54		0.62	

For the individual breath sample datasets, the mean classification accuracy for the different methods was similar and ranged from 66.5% to 68.3% with no statistical difference between the different breaths. An optimum accuracy of 70.7% was obtained using the JRIP classifier for breath 1. This difference is statistically significant when compared with J48 and PART classifiers. The same is true for the result for breath 2. Classification accuracy was improved by combining breath samples into datasets. Combining two of the breath samples in various combinations (1&2, 1&3 and 2&3) gave mean accuracies between 70.8% and 73.0%, with an optimum (but statistically equivalent) accuracy of 73.7% achieved using breaths 1&2 with the J48 classifier. It is known that J48 is an optimistic classifier [27], and indeed this is reflected in the high standard deviation of the classification accuracies when compared with other learners. This is also supported by the outcome of the significance test which shows that although the absolute classification accuracies for J48 are higher, the gains are not statistically significant. When breaths 1, 2 and 3 were summed, the mean classification accuracy for the different methods was 73.4%, with an optimum accuracy of 74.1% achieved again with the J48 classifier learner. The standard deviation for the accuracies was noticeably lower when data from two breaths were combined into single data sets; though, this was less significant when all breaths were summed. Overall, the JRIP classifier was most consistent. The J48 classifier tended to the most unstable, with highest standard deviations relative to the mean. This was particularly true for individual breaths where there were fewer objects to learn from. However, for the combined datasets there was little to separate the three different learners.

Mean AUC values were between 0.64 and 0.67 for individual breaths, while breath 1 and 2 was the best scoring breath combination with a mean AUC of 0.70 though this difference was not statistically significant. A similar pattern was observed in terms of stability; with J48 the most unstable, and JRIP the most stable and consistent in terms of AUC value regardless of the data used. The proportion of correctly classified COPD patients (true positives) ranged from 70% to 80% which was greater than the proportion of control subjects correctly identified at 52% to 62%. The pattern of stability for the correctly classified COPD subjects differed to that of the previous observations; although J48 performed poorly for two of the single breaths, JRIP (the most stable learner in terms of overall classification accuracy) also tended to suffer from instability in this respect. JRIP also gave some of the lowest levels of control subjects correctly identified.

### 3.3. Discussion

A substantial variation was found in the levels of VOC in breath during three repeat samples over a short time period. The extent of the variation in VOC levels differed between COPD and healthy subjects and the patterns of variation differed for isoprene *versus* the bulk of other VOCs. Different breath repetitions were similar in their ability to act as a discriminator for COPD *versus* healthy states and aggregation of three individual breath samples into single data sets for each subject improved classification accuracy (and stability). Reasons for this variation and the implications for breath testing protocols are explored in this section.

#### 3.3.1. Variation in Isoprene

Isoprene is ubiquitous and also one of the most highly concentrated VOCs in breath. It is an endogenous not environmental VOC linked to cholesterol metabolism [28]. In this study, levels were found to fall steadily for both COPD and control groups with repeat breath sampling. A reduction with sequential breaths might be accounted for by progressive clearance of the alveolar air, containing mainly endogenous VOCs, through repeated full exhalation before equilibrium with the VOCs from blood can occur in the alveolus. However, a similar pattern of variation was not observed with total VOC concentrations (or any other VOCs found at high frequency), suggesting different mechanisms for breath isoprene expression than other VOCs. Exhaled isoprene has been measured over the short term, within individuals, using continuous monitoring methods. Concentration of breath isoprene has been demonstrated to vary with exertion levels [29,30,31]; with the isoprene content of exhaled air increasing dramatically at the start of exercise followed by a steep decline to below the initial level. For example, King *et al*. [30,31] found that isoprene peaked about one minute into moderate exercise with levels up to 3–5 times higher than rest levels and propose that isoprene variation is due to varying heart and exhalation rates but not due to variation in endogenous production. The high volatility and low solubility of isoprene, allows it to readily evaporate into the lungs. Karl *et al*. [29] propose that at the onset of exertion, as blood flows through the lungs at a higher speed, there is greater evaporation of blood isoprene into the lungs, initially causing higher breath isoprene, then a subsequent drop in blood isoprene due to this loss by evaporation, leading to a decrease in breath levels. This is then exacerbated by an increase in breath rate which dilutes isoprene further. King *et al*. suggest that isoprene might have potential for assessing changes in hemodynamics, pulmonary function and gas exchange [31].

The level of exertion during the sampling in this study is small but pronounced variation in isoprene has been reported during the sleep cycle where only modest variations in gas exchange occur (when compared with exercise regimes) [29,32,33,34]. Amann reported that spikes in heart rate were accompanied with doubling of breath isoprene [33]. Intra-subject isoprene variation, in terms of coefficient of variation, was comparable to that reported by Barker *et al*. [4] who reported an average of 21% variation in isoprene per subject, albeit over 15 min sampling intervals in a study of cystic fibrosis patients and healthy subjects. The methods employed in this study are not continuous, and will not therefore capture the full pattern of VOC variation due to sampling, but it is proposed that during the sampling period, there is an increase in pulmonary ventilation and blood flow which increases isoprene clearance from the blood. This clearance is greater in isoprene than in other VOCs due to the high volatility but low affinity for blood. There is also the additional “wash-out” effect, as alveolar air is replaced with inspired air with very little isoprene. Proportionally, there was a greater reduction in isoprene in sequential breaths in the healthy control group and this is the first study to report less reduction in isoprene in sequential breaths in people with COPD. This might be because healthy subjects can inhale and particularly exhale more fully than those with COPD, and clear isoprene from the alveolar region of the lungs. The way in which breath isoprene is expressed must be taken into account if it is to be used as a distinguishing VOC in diagnostic or monitoring tests; as these variations can potentially disguise or indeed exacerbate apparent variation due to disease state. 

#### 3.3.2. Variation in Total and Other VOCs

Between breath samples 1 and 2, there was an increase in total VOC in the COPD group but little change for the healthy controls. For both groups however there was a decrease in total VOC in breath 3. The mechanism of increased replacement of alveolar air with inspired room air due to increased pulmonary vascular hemodynamics (“wash-out”), would suggest a continued fall in total VOCs across the sequential breaths rather than increased or static levels. The rise in breath delivery in the second attempt in the COPD group could be due to a practice effect (e.g., better mouth seal around the sampler) or possibly deeper exhalation with more subject confidence. Furthermore, the ability to sustain a deep expiration will be more difficult in people with lung disease, which will affect expression of breath VOC in the current sample and wash-out levels for the following sample. A greater subject effort was observed by the investigator in both groups, but especially COPD in the second and third breath samples than in the initial sample, where confidence was sometimes low. This suggests an exercise, or learning effect, or compensation for poor initial exhalation with stronger exhalation in repeat samples. Benzene and toluene, examples of other ubiquitous VOCs which contribute towards the total, displayed similar variability in this study to that reported by Barker *et al*. [4]. Again, as with isoprene, variations in these levels will influence the outcome of diagnostic tests although the variation in the individual VOCs should be less pronounced than that seen in isoprene.

A range of VOCs are mentioned in this study. Of these, benzaldehyde, benzene and toluene are likely to be due to environmental factors, including but not limited to inhaled cigarette smoke, rather than being produced by metabolism. These need to be differentiated from VOCs more likely due to metabolic processes, such as isoprene and linear alkanes and aldehydes as their levels may be linked with short-term exposure events rather than disease. The contribution of smoking to processes such as oxidative stress should also be considered and ideally markers of COPD related inflammation should be isolated. Jareno-Esteban *et al*. [35] propose that certain aldehydes are potentially useful indicators of smoking related oxidative stress which are not due to cigarette smoke itself. Fens *et al*. [36] link a range of VOCs with indicators of COPD related inflammatory activity in sputum, namely eosinophilis and primarily neutrophilis. These included branched, unbranched and cyclic alkanes, benzene, toluene, and alkylated benzene, the only component linked to both indicators. However many of these compounds are also linked to smoking.

#### 3.3.3. Relative Ability of Breath Repetitions to Discriminate COPD and Healthy States

Despite the variations in VOC levels in sequential breaths and the differing patterns in this variation between COPD patients and healthy controls noted previously, the breath repetitions (1, 2 and 3) gave similar classification accuracy to one another when using the three classifier methods detailed previously. It should however be noted that this was not the case with other less robust classifiers (see supplementary data). Aggregating breath data into single data sets improved classification accuracy further and reduced the variability between the FCV runs as indicated by the lower standard deviations. Breath VOC data is typically prone to outliers and aggregation of the data objects (breath samples) mitigates noise in the data, to some extent, by summarizing the contribution of VOCs and helping to reduce the number of individual VOC objects relevant for each patient. This depends on the type of aggregation employed and indeed on the classifier used (supplementary data). The use of maxima from the three breaths, for example, gave poor results for the less robust classifiers. The second breath generally yielded the most data for the COPD group and the greatest difference in total VOC magnitude between COPD and control groups, but it did not give the optimal classification accuracy. However, the second breath also appeared to be most affected by noisy data and outliers; giving more extreme low scores in classification accuracy in the less robust classifiers (see supplementary data). Breath 3 had the lowest amount of VOC, and breath 1 an intermediate amount and both were more reliable for these classifiers.

Classification accuracy was poor when individual breath data were used (breaths 1, 2, 3) and higher scores have been obtained previously by treating all three breaths as separate objects in the same analysis [16]. Combining samples improved matters, but did not result in a classification accuracy that could be used for practical testing. For this reason the results should be considered preliminary and subject to follow-on trials with improved VOC capture. Better matching of smoking status between the groups should generally improve classification accuracy; although this reduces the number of objects which are available to learn from even further. Furthermore, finding elderly heavy smokers without physiological lung obstruction is difficult. Although experimentation was carried out by removing current smokers from the dataset, it was not found to be beneficial for the aggregated data as it further skewed the class imbalance towards COPD patients. Learning approaches often suffer from bias towards the majority class and thus, offer poor prediction for the minority class(es). This would explain the poor prediction performance for the control subjects (Table 3). This needs to be considered at the subject recruitment stage; with controls matched for smoking status and other likely confounders as far as possible. It should be noted that the data for this study has an inherent class imbalance, *i.e.*, the COPD patients are better represented than healthy controls. 

#### 3.3.4. Implications

Variations in VOC levels due to the sampling procedure need to be isolated from variation in endogenous synthesis or indeed re-expression/metabolism of exogenous VOCs. The findings of this study should be transferable to other sampling methods such as the use of Tedlar bags, for which exhalation rate is not controlled, and should also be affected by subject factors. There is a need to consider exertion history prior to sampling which could include practice breaths, spirometry, duration of the rest period, exertion due to walking to the test room *etc*., as these might have an effect on apparent exhaled VOCs and hence discrimination; which should apply regardless of sampling method. These factors should, as far as possible, be integrated into the sampling protocol, as these may have a short term effect on VOC levels, with cardiovascular and respiratory monitoring to assess their role. Dynamic analysis of breath, rather than stand-alone sampling can further contribute towards the understanding of the mechanisms behind lung disease and response to exertion.

In addition to the effort of the subject, the amount of time between breath sampling and reacquiring equilibrium between the blood stream and alveolar air is not currently known; it will be dependent on the individual subject, the effort made in previous breaths and the disease state, and requires further study. Any technique that relies on absolute levels of particular VOCs to distinguish disease state will be affected by this.

#### 3.3.5. Improvements to Current Techniques

Increasing sample volume and the sensitivity of the detection equipment should yield improvements, as more low concentration VOCs can be detected. Combining multiple samples does generate better data for the subject by effectively increasing the volume of breath sampled. The sampler used in this study has a smaller volume than, for example, the Tedlar bags used in other studies [8]. There is also the issue of the composition of the breath in the sampler (lower or upper airway). The BioVOC sampler does not control this, per se, and it is reliant on both subject effort and ability in order to displace lower airway air as exhalation proceeds. Automated methods to control alveolar air collection, such as exhaled carbon dioxide monitoring [37], or rebreathing circuits [6] might improve matters but this must be balanced against their practicality in the clinical environment. Online “real-time” systems [30] are not currently suitable for measuring the full range of breath VOCs and can only be used to target small numbers of compounds at a time.

Finally, as more becomes known about biosynthesis and elimination pathways of VOCs, the relevance of VOCs as markers for certain diseases can be put into context and VOCs can be used as diagnostic and monitoring tools within a clinical setting. Simulation and modeling of hemodynamics and pulmonary function during VOC testing will provide clues for interpreting VOC profiles and are vital to developing an objective test for use in a clinical environment.

## 4. Conclusions

VOC levels varied during three sequential breath samples. The extent of the variation in VOC levels differed between COPD and healthy subjects and the patterns of variation differed for isoprene *versus* the bulk of other VOCs. The three breath samples gave similar classification accuracy to one another when evaluated separately with 66.5% to 68.3% of subjects classified correctly depending on the breath repetition used. Aggregating multiple breath samples into single data sets gave noticeably better discrimination than data from single breaths, with classification accuracy improving to 73.4%, with increased classifier stability. Accuracy is not yet sufficient for reliable diagnosis against current clinical standards but could be improved by enhanced sampling and analysis. However, this study has shown that minute-by-minute variability in VOC samples, and short-term effects of practice or exertion are factors that should be considered in any breath testing program, such that samples are more representative and offer optimal discriminatory characteristics.

## Supplementary Files

Supplementary File 1 Supplementary File (PDF, 136 KB)
