# Supplementary material for: Short-Term Intra-Subject Variation in Exhaled Volatile Organic Compounds (VOCs) in COPD Patients and Healthy Controls and Its Effect on Disease Classification
Source: Metabolites. 2014 May 9;4(2):300–18. doi: 10.3390/metabo4020300 (PMC4101508; doi:10.3390/metabo4020300)
Supplement: Supplementary File 1 — Supplementary File (PDF, 136 KB) [file metabolites-04-00300-s001.pdf]

Supplementary File.

Additional Data for Short-Term Intra-Subject Variation in Exhaled Volatile Organic Compounds (VOCs) in COPD Patients and Healthy Controls and Its Effect on Disease Classification

Data is based on 10 fold cross-validation.

Table S1. Additional classifier learning methods used in the study.

Classifier Learner	Description
SMO (Polykernel)	Sequential minimal optimisation approach for support vector machines [1]
FuzzyNN	Fuzzy nearest-neighbour classifier [2]
Random Forest	A tree-based classifier [3]
FRNN	A fuzzy-rough set nearest-neighbour approach [4]
VQNN	A noise-tolerant fuzzy-rough set-based classifier [5]

Table S2. Classification accuracy for individual breath samples.

Classifier	Breath 1				Breath 2				Breath 3			
	Overall%	COPD%	Control%	AUC	Overall%	COPD%	Control%	AUC	Overall%	COPD%	Control%	AUC
J48	76.7	76.0	78.3	0.83	64.4	66.0	60.9	0.58	65.8	76.0	43.5	0.59
JRIP	75.3	78.0	69.4	0.76	72.6	82.0	52.2	0.67	58.9	68.0	39.1	0.54
PART	71.2	76.0	60.0	0.74	68.5	74.0	56.5	0.67	58.9	64.0	47.8	0.57
SMO	53.4	54.0	52.2	0.53	60.3	64.0	52.2	0.58	52.1	60.0	34.8	0.47
FuzzyNN	57.5	56.0	60.9	0.58	53.4	60.0	39.1	0.50	57.5	64.0	43.5	0.54
Random Forest	67.0	80.0	39.1	0.61	71.2	78.0	56.5	0.72	65.8	78.0	39.1	0.68
FRNN	46.6	48.0	43.5	0.39	37.0	8.0	100.0	0.56	56.2	62.0	43.5	0.55
VQNN	35.6	10.0	91.3	0.38	32.9	2.0	100.0	0.53	45.2	30.0	78.3	0.56

Table S3. Classification accuracy using **mean** aggregation.

Mean	Unreduced Data		
Classifier	Overall%	COPD%	Control%
J48	74.3	81.0	61.7
JRIP	72.4	77.9	62.2
PART	71.9	79.0	58.5
SMO	71.8	84.3	48.3
FuzzyNN	63.1	86.2	19.7
Random Forest	70.0	89.7	33.2
FRNN	36.4	7.3	90.9
VQNN	34.8	0.0	100.0

Table S4. Classification accuracy using **sum** aggregation.

**Sum**	**Unreduced Data**		
**Classifier**	**Overall%**	**COPD%**	**Control%**
J48	74.2	80.8	61.9
JRIP	73.3	78.3	64.0
PART	72.7	79.3	60.4
SMO	71.8	84.3	48.3
FuzzyNN	63.1	86.2	19.7
Random Forest	68.8	88.3	32.3
FRNN	36.4	7.3	90.9
VQNN	34.8	0.0	100.0

Table S5. Classification accuracy using **maximum** aggregation.

**Maximum**	**Unreduced Data**		
**Classifier**	**Overall%**	**COPD%**	**Control%**
J48	65.6	74.0	49.8
JRIP	70.6	78.3	56.3
PART	67.5	77.2	49.3
SMO	70.5	84.6	44.2
FuzzyNN	62.7	82.3	25.9
Random Forest	71.8	91.4	35.1
FRNN	37.3	8.4	91.5
VQNN	35.7	1.3	100.0

Table S6. Classification accuracy using **minimum** aggregation.

**Minimum**	**Unreduced Data**		
**Classifier**	**Overall%**	**COPD%**	**Control%**
J48	66.6	74.5	51.7
JRIP	67.6	79.7	44.8
PART	65.5	73.1	51.3
SMO	69.3	83.8	42.1
FuzzyNN	65.6	83.7	31.6
Random Forest	71.0	87.5	40.2
FRNN	64.9	66.5	61.9
VQNN	65.7	67.1	63.0

Table S7. Classification accuracy using **ordered weighted** aggregation (OWA).

**OWA**	**Unreduced Data**		
**Classifier**	**Overall%**	**COPD%**	**Control%**
J48	75.2	81.8	62.8
JRIP	73.6	78.7	64.0
PART	72.5	79.8	58.8
SMO	71.5	84.4	47.4
FuzzyNN	63.1	85.2	21.6
Random Forest	69.9	88.9	34.2
FRNN	36.4	7.1	91.3
VQNN	34.9	0.2	100.0

References

1. Zadeh, L.A. Fuzzy sets. *Inform. Contr.* **1965**, *8*, 338–353.
2. Aha, D.; Kibler, D. Instance-based learning algorithms. *Mach. Learn.* **1992**, *6*, 37–66.
3. Breiman, L. Random Forests. *Mach. Learn.* **2001**, *45*, 5–32.
4. Jensen, R.; Cornelis, C. A New Approach to Fuzzy-Rough Nearest Neighbour Classification. In Proceedings of the 6th International Conference on Rough Sets and Current Trends in Computing, Acron, OH, USA, 23–25 October 2008; pp. 310–319.
5. Jensen, R.; Cornelis, C. Fuzzy-Rough Nearest Neighbour Classification. In *Transactions on Rough Sets XIII*; Lecture Notes in Computer Science Volume 6499; Springer: Berlin/Heidelberg, Germany, 2011; pp 56–72.

© 2014 by the authors; licensee MDPI, Basel, Switzerland. This article is an open access article distributed under the terms and conditions of the Creative Commons Attribution license (http://creativecommons.org/licenses/by/3.0/).
